# Evaluating Spatial Accessibility to General Hospitals with Navigation and Social Media Location Data: A Case Study in Nanjing

**DOI:** 10.3390/ijerph17082752

**Published:** 2020-04-16

**Authors:** Tianlu Qian, Jie Chen, Ang Li, Jiechen Wang, Dingtao Shen

**Affiliations:** 1 Jiangsu Provincial Key Laboratory of Geographic Information Science and Technology, Key Laboratory for Land Satellite Remote Sensing Applications of Ministry of Natural Resources, School of Geography and Ocean Science, Nanjing University, Nanjing 210023, China; qiantl@smail.nju.edu.cn (T.Q.); Angli@smail.nju.edu.cn (A.L.); wangjiechen@nju.edu.cn (J.W.); 2 School of Architecture and Surveying Engineering, Datong University, Datong 037009, China; chenjie_301@126.com; 3 Jiangsu Center for Collaborative Innovation in Geographical Information Resource Development and Application, Nanjing University, Nanjing 210023, China; 4 Changjiang River Scientific Research Institute, Changjiang Water Resources Commission, Wuhan 430010, China

**Keywords:** accessibility, general hospitals, equity, gaps between traffic modes, navigation data, population spatialization with social media data

## Abstract

Spatial accessibility to general hospitals is an important indicator of the convenience and ability of residents to obtain medical services. Therefore, developing a model for measuring accessibility to general hospitals by multiple transportation modes is necessary. In this study, considering that the increase in travel time will reduce the attractiveness of general hospitals, we used the Two-Step Floating Catchment Area with the Gaussian attenuation function, in which the supply was presented by capacity of hospitals (i.e., number of beds), and the demand was presented by population in each grid derived with social media data mapping real-time locations of active users. The Gaussian Two-Step Floating Catchment Area (Ga2SFCA) simulates the attenuation tendency of the general hospital service capabilities over transit time. To obtain a highly precise understanding of accessibility to hospitals, transit time on Baidu Maps’ navigation service was used as the impedance condition, and the study area was divided into 1 square kilometer grids as the basic unit of research. Taking Nanjing city as a case study, it is found that the accessibility distribution shape changes from a multi-centered circular pattern to a multi-peak distribution, as the time threshold increases. By comparing the accessibility among 11 districts varying from main urban area to suburbs, the accessibility to general hospitals in Nanjing is significantly regionally unbalanced in both travel modes. By calculating and mapping the Modal Accessibility Gap (MAG) of the two travel modes, different modes of transportation resulted in different general hospital accessibility distributions. Generally, private car is superior in access to general hospitals to public transit in most areas. In the central area, public traffic may not contribute to the access to medical services as much as we thought, rather it plays a role in areas far from hospitals along metro lines and bus routes.

## 1. Introduction

Public service facilities are a basic requirement for the survival and development needs of citizens, and are an important part of the daily life of residents. Since the 1980s, many scholars have studied accessibility in terms of the spatial layout of public service facilities (schools, hospitals, shopping centers, etc.) [1,2,3,4,5,6]; these studies use accessibility results to assess the equity of social and economic activities, as well as the balance of public service resource allocation within cities. General hospitals are one of the most important public service facilities because they safeguard peoples’ health and lives. The layout of general hospitals is directly related to the equitable and efficient allocation of medical resources by the government, as well as the quantity and quality of medical services enjoyed by the residents.

Hansen proposed the concept of accessibility in 1959 after several years of research and development, and since then, it has been applied in many fields [7]. Accessibility is affected by various factors. Spatial accessibility emphasizes the spatial attributes of accessibility (travel time/distance cost, transportation mode, and land use) but ignores the nonspatial factors (personal preference, religion, and social position). Therefore, spatial accessibility is usually defined as the ease with which residents in a particular area can reach a particular service or facility, reflecting the ease with which residents can overcome space or time impedances to access facilities; it can intuitively measure the balance of the layout of public service facilities and reflect whether residents can access public services fairly from a spatial prospective [8]. Many scholars have conducted related research on methods of measuring accessibility, including the measurement method of accessibility, the calculation framework of accessibility, and the evaluation model that combines accessibility with land use, transportation systems, subjective decision-making, and so on [9,10,11,12]. Among these methods, the Two-Step Floating Catchment Area (2SFCA) has been widely studied and applied in the field of urban public service facilities because it is better able to account for the ratio between the supply and demand sides. Radke first proposed the 2SFCA method, which was improved and named by Luo et al. [13,14]. 2SFCA provides services simultaneously for multiple supply facilities that meet the threshold conditions, not only calculating the supply–demand ratio of the supply facility but also quantifying the availability of the demand object. In recent years, an increasing number of scholars improved the 2SFCA method in studies on the following types of public service facilities: medical facilities [15,16,17], emergency shelters [18], schools [19], parks [20,21], food deserts [22,23], and parking lots [24]. General hospitals are important medical service facilities in urban cities, so comprehensively evaluating the accessibility to general hospitals needs to take into account the supply–demand ratio and travel distance/travel time impedance. Therefore, the 2SFCA method is suitable for assessing accessibility to general hospitals.

However, there have been two major issues with previous accessibility research based on the 2SFCA method. On one hand, reliable travel time is the key to achieving a more reliable analysis of the accessibility score. Most previous studies established road network topology to obtain the travel distance or travel time between the origin and ending location. The results calculated by this scheme have some limitations, which include the difference in the driving speed set based on the road level and actual driving speed, and problems with path analysis depending on the accuracy of the road network data. On the other hand, most previous studies assume that users get to facilities by the same mode of transportation (usually by car) [25]. From the aspect of demand, urban residents prefer multi-mode rather than single-mode transportation, especially for healthcare service demand with varying intensity and mobility for different sections of the population. In general, both the elderly and minors have higher healthcare service demand and intensity than others and are more dependent on public transportation because of the restrictions of car licensing and driving ability [26]. Therefore, evaluation of the accessibility to general hospitals simply by establishing a road network topology to simulate private car travel is highly limited. Also, “Nanjing 2018–2020 Medical and Health Service System Planning” pointed out that the imbalance of medical and health resource allocation is one of the main contradictions in the construction of the medical and health service system in Nanjing [27].

With the wide application of geographical information science, location-based services allow population data to be acquired at both high temporal and high spatial precision. Social media data is one of the most popular sources owing to its wide range of users. Many researches focused on delineating population distribution or urban structure with social media data. Long and Shen [28] spatialized population with online check-in, POIs (Points of Interest), and other open data. Gao et al. [29] detected city community network with interaction and movement of cell phone users. Chen et al. [30] found urban function areas with building-level social media data. Locations of individuals are essential to urban structures and associate with medical service demand.

To explore the accessibility of general hospitals by different transit modes, this paper introduces the Gaussian Two-Step Floating Catchment Area (Ga2SFCA) as the accessibility calculation model. To evaluate up-to-date demand of medical service, social media data helps to determine the population spatialization. Population is gridded to 1 km cells to finely delineating the demand. Sourcing from open map navigation data, travel time used in this paper is in line with reality and is more reliable than simply simulating travel time on road network, additionally offering different travel times with various transportation modes. The study aims to explore the spatial distribution of and differences in accessibility to general hospitals in Nanjing by the private car and public transit modes from a higher resolution level and to provide a detailed reference for improving the allocation of medical resources among different segments of the population accessing general hospitals.

## 2. Materials and Methods

In the field of hospital accessibility assessment, Higgs first introduced the number of hospital beds, the number of medical personnel, traffic conditions, and population density as evaluation factors [31]. Similarly, we used the number of beds as a hospital’s ability to provide medical services, used travel time as the impedance condition for residents to access hospitals, and used the population as the grid demand.

### 2.1. Study Area

**Figure 1:**
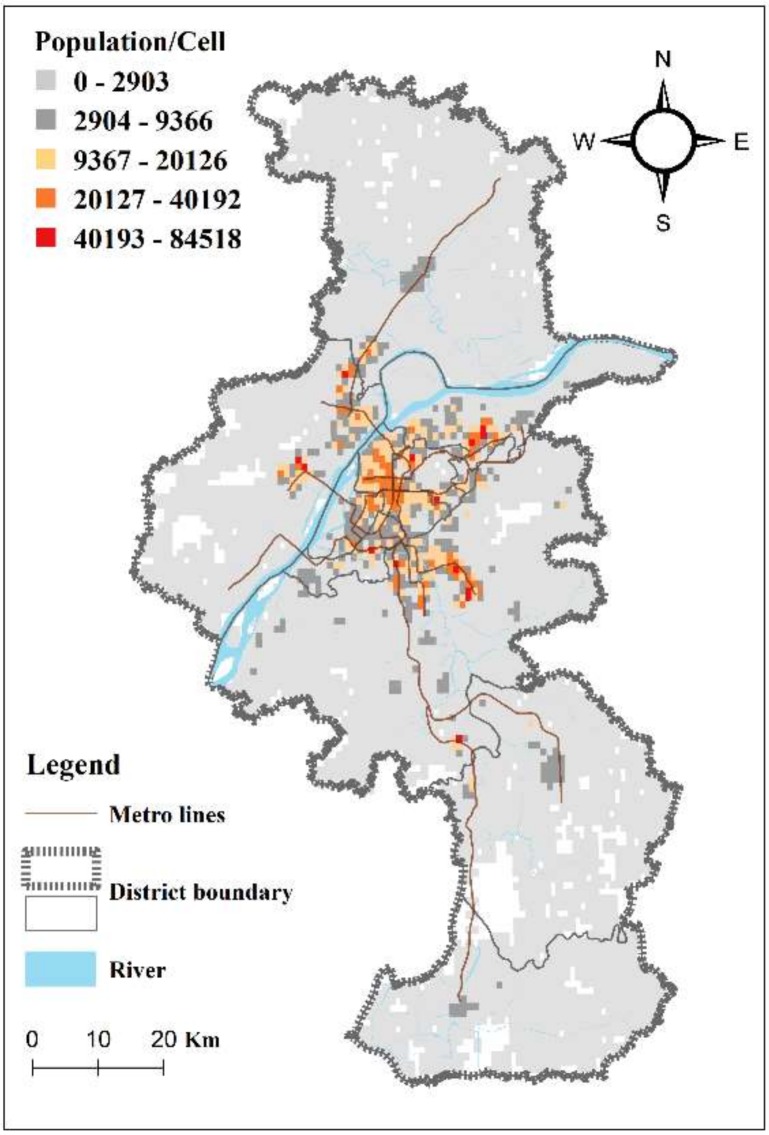
Population density in Nanjing derived using Kernel Density Evaluation (KDE) with EasyGo dataset of Tencent, a social media data mapping real-time location of users on their cell phone.

**Figure 2:**
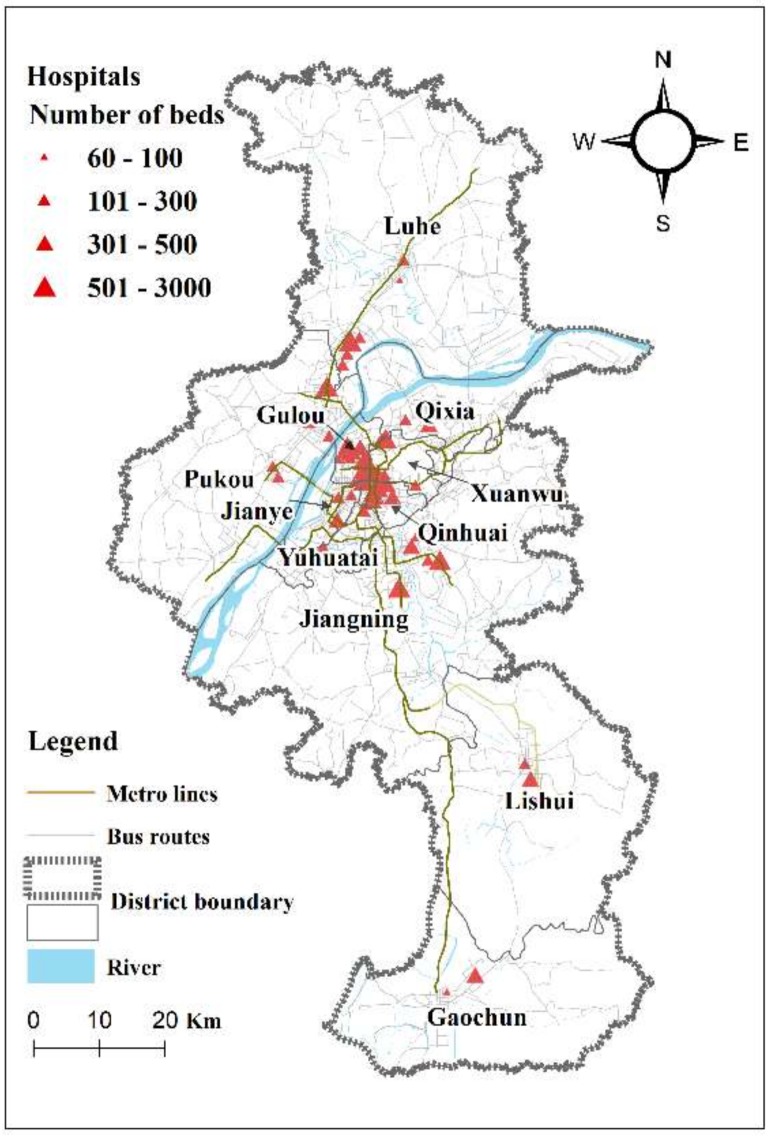
General hospitals in Nanjing symbolized by red rectangles, with sizes of rectangles representing numbers of beds.

Located in the Yangtze River Delta, Nanjing lies at 31°14’–32°36’ N, 118°22’–119°14’ E. Nanjing is the capital of Jiangsu Province, the economic and cultural center of Jiangsu Province, and an important city in eastern China. The urban area covers 6622 square kilometers, with a resident population of 8.33 million inhabitants. Nanjing’s urban public transit system has developed rapidly. By the end of 2017, there were more than 700 operating bus lines and more than 8000 operating vehicles. The Nanjing Metro has opened nine lines—totaling 347 km in length—with 164 stations that cover 11 districts of Nanjing. Nanjing has become one of the cities in China where all districts are serviced by the Metro (Figure 1). As of the end of 2017, according to Nanjing’s land use planning administration, Nanjing’s urban area is divided into 11 administrative districts: Gulou, Xuanwu, Qinhuai, Jianye, and Yuhuatai districts jointly constitute the main urban area of Nanjing. Qixia, Jiangning, and Pukou districts are the vice urban area of Nanjing. Luhe, Gaochun, and Lishui are three suburbs of Nanjing [32] (Figure 2).

### 2.2. Data Source

#### 2.2.1. Population Data

Previous accessibility studies mostly used sub-districts or residential areas as research units to ensure that population data matched those areas. However, this kind of scheme has certain limitations. On the one hand, it is necessary to select a centroid point as representative of the residential area or sub-district, which means that the spatial difference of accessibility within the residential area or sub-district cannot be displayed. On the other hand, a residential area or sub-district is usually a large geographic unit, which has the disadvantage of low spatial resolution that cannot fully reveal the spatial distribution and difference. A powerful way to improve the resolution of urban structure analysis is to divide the urban study area into a regular grid [33]. Therefore, this study uses a square grid (1000 m × 1000 m) as the basic cell to measure the population data of Nanjing, allowing the demand of medical services to be delineated in a fine spatial resolution.

The population grid was derived from EasyGo dataset of Tencent, one of the largest social media provider in China with more than a thousand million active users. This dataset is produced by mapping real-time locations of active users who are using Tencent apps with location-based services on their cell phones. We acquired EasyGo data in Nanjing for an entire week from 17 to 23 October. In our accessibility calculations, the population of each square grid represents its demand indicator for medical services. To map the housing population density as a representative medical service demand, the dataset was acquired in off-peak hours ranged from 11 p.m. to 1 a.m.

To calculate the population density of each grid with EasyGo data, Kernel Density Evaluation (KDE) was introduced. We calculated kernel density with a search radius of half of the grid side (500 m) and using average density within a grid as the grid density. Nanjing has a residential population of about 8.33 million. Therefore, the population density of each grid was proportionally increased. Each grid has a maximum population of 84,518 and a minimum of 0. The population density of Nanjing is shown in Figure 1.

#### 2.2.2. General Hospital Services Overview

Based on 2014 data, the medical service facilities in this study include 56 general hospitals in Nanjing (excluding specialist hospitals that perform only one branch or several branches of medicine, such as eye hospitals, children hospitals, cancer hospitals, mental hospitals, rehabilitation nursing hospitals, etc.). Of these 56 general hospitals, there are 29 secondary hospitals with a total of 6204 beds and 27 tertiary hospitals with a total of 19,059 beds; the total population of Nanjing is about 8.33 million, and thus the number of beds per thousand people is 3.05. The locations of the hospitals are shown in Figure 2. The symbol reflects the number of beds in each hospital.

#### 2.2.3. Travel Time

**Figure 3:**
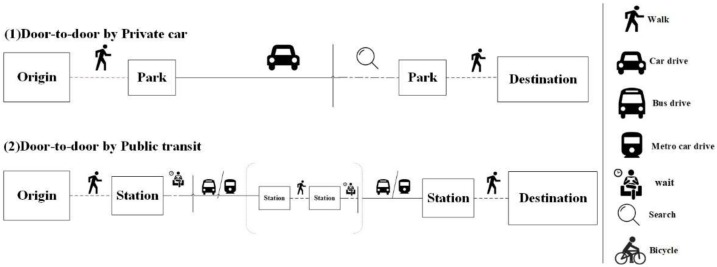
Door-to-door approach to calculate complete travel time for (1) private car journey, (2) public transit journey.

This study develops a data crawling tool that is based on Baidu Maps’ navigation service Application Programming Interface (API) to harvest the travel time of residents using both private car and public transit to reach the closest hospital [34]. Baidu Maps’ navigation service is supported by massive high-precision road network data, which integrate dynamic traffic information and provide a door-to-door approach (Figure 3) to calculate the complete navigation phase for various modes of transportation (private car, public transit, cycling, and walking). This method of obtaining travel times not only overcomes the defect that travel time calculated on the basis of road network topology often varies substantially from actual travel time, but also allows travel times to be updated frequently, thereby improving the reliability of travel time. On the other hand, as stated above in Section 1, multi-mode transportation better accords with the fact of urban residents. Travel time for various modes of transportation would make the results of this study more practically significant. For medical facilities of which demand varies for different sections of population, it is also essential to evaluate accessibilities in different transportation modes. In this study, the origins input into the navigation service are the centroid of each cell, and the destinations are the 56 general hospitals in Nanjing. Using the 6777 square grids (1000 m × 1000 m) of the study area, we calculated the travel time from the centroid of each cell to all 56 hospitals during the off-peak hours by private car and public transit modes. Thus, we calculated 759,024 travel time costs (2 × 56 × 6777). The results show that the average travel time in a private car is 57 min, the minimum travel time is 1 min, the maximum travel time is 415 min, and the average travel time within which all residents access the nearest hospital is 23 min. By public transit, the average travel time is 160 min, the minimum travel time is 1 min, the maximum travel time is 1127 min, and the average travel time within which all residents access the nearest hospital is 80 min. It is clear that the average time consumed by public transit is about 2.81 times more than by private car, which indicates that the impact of transportation modes on accessibility is significant. This confirms that the exploration of accessibility differences between private and public transportation is necessary.

### 2.3. Methods

**Figure 4:**
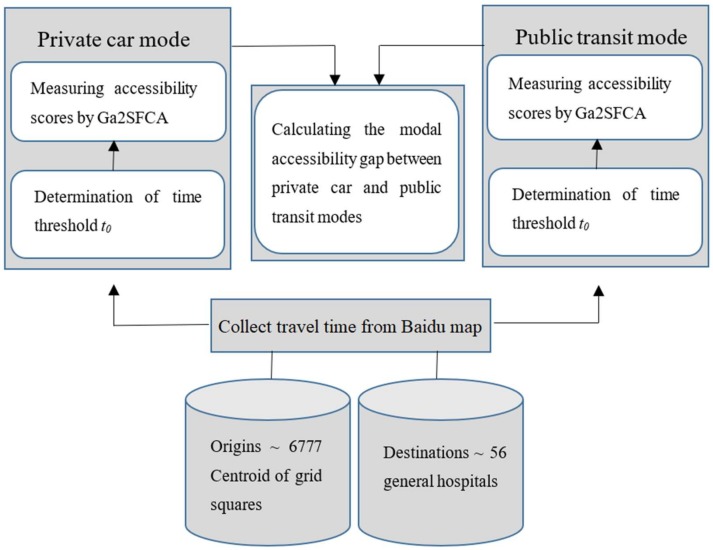
Workflow of the accessibility calculation model, the Gaussian Two-Step Floating Catchment Area (Ga2SFCA).

Ga2SFCA is the accessibility calculation model in this paper. This model uses the travel time for private car and public transit modes harvested from an internet map-based navigation service as the path analysis and measures the Modal Accessibility Gap (MAG) to evaluate the spatial distribution of differences in the private car and public transit modes of accessibility to general hospitals. The workflow of Ga2SFCA is shown in Figure 4.

#### 2.3.1. Ga2SFCA Based on Internet Map’s Navigation Service

Scholars have made a series of improvements to 2SFCA to reinforce its applicability to various cases. The main improvements to the 2SFCA method relate to the fact that different distance attenuation functions have different attenuation trends. These modified methods can be roughly classified into three types: (1) Enhanced 2SFCA (E2SFCA), which introduces a distance attenuation function based on 2SFCA and segments the distance within the search radius [35]; (2) Kernel Density 2SFCA (KD2SFCA), which is based on 2SFCA and adds a continuous kernel density form of distance attenuation function within the search radius [18]; and (3) Gaussian 2SFCA (Ga2SFCA), which uses a Gaussian function as the distance attenuation function within the 2SFCA search radius [36].

Ga2SFCA was first proposed by Dai [35]. The essence of Ga2SFCA is to add an “anti-S” shaped Gaussian distance attenuation function to the cell within the search threshold range on the 2SFCA method, so that the change of accessibility with distance attenuation appears as a “slow–fast–slow” rate. Therefore, Ga2SFCA not only provides the search threshold of supply and demand cells, but also calculates the influence of spatial distance attenuation on supply and demand ratio. The specific steps this study employed to calculate the spatial accessibility of general hospitals in Nanjing using Ga2SFCA are as described below [14,36].

First, at each general hospital location *j*, the time threshold t_0_ for the medical service supply range is determined, and each population cell *k* (square grid centroid) within the t_0_ search range is given a corresponding weight by the Gaussian attenuation function. All of the weighted cells within the search range are summed and then negotiated with the supply scale of the hospital *j* to obtain the supply–demand ratio R_j_ of the hospital *j*. The R_j_ is calculated by the following Equation (1):

(1) $$R_{j}=\frac{S_{j}}{\sum_{k\in \left\{t_{kj}\leq t_{0}\right\}}g(t_{kj},t_{0})P_{k}}$$

In Equation (1), *S_j_* represents the number of beds in the hospital, *P_k_* represents the cell population within the search threshold, *t_kj_* represents the travel time between population cell *k* and hospital *j*, and *g(t_kj_, t_0_)* is the weight of the cell *k*, which can be further expressed as the Gaussian attenuation function. Equation (2) is as follows:

(2) $$g(t_{kj},t_{0})=\left\{\begin{array}{c} \frac{e^{-1/2\times {\left(t_{kj}/t_{0}\right)}^{2}}-e^{-1/2}}{1-e^{-1/2}},t_{kj}\leq t_{0}\\ 0,t_{kj}>t_{0} \end{array}\right.$$

In Equation (2), each variable has the same meaning as Equation (1). Equation (2) follows the common form of Gaussian attenuation function, in which the exponent forms a bell curve to simulate the demand for medical services decaying with travel time increasing.

Second, for each population cell centroid *i*, the time threshold t_0_ for the medical service demand range is determined. Similarly, the Gaussian attenuation function is used to assign a corresponding weight to the supply–demand ratio of each supply hospital in the *t_0_* search range, and the weighted supply and demand ratio of all hospitals in the search range is summed to obtain the medical service accessibility of the population cell centroid *i*. The $A_{i}$ is calculated by the following Equation (3):

(3) $$A_{i}=\sum_{j\in \left\{t_{\mathrm{ij}\leq }t_{0}\right\}}g\left(t_{\mathrm{ij}}{,t}_{0}\right)R_{j}$$

In Equation (3), $t_{\mathrm{ij}}$ represents the travel time between population cell *i* and hospital *j*, and ${g(t}_{\mathrm{ij}}{,t}_{0})$ is the weighted value of the hospital *j* service capability calculated by Equation (2). The magnitude of the $A_{i}$ value reflects the degree of time and space impedances to acquiring the medical service for the population cell. A larger $A_{i}$ value indicates that the barrier to accessing medical services is lower.

#### 2.3.2. MAG between Private Car and Public Transit Modes

In order to directly show the spatial distribution of differences in private car and public transit modes of accessibility to general hospitals, the Modal Accessibility Gap (MAG) was introduced into our model. Kwok and Yeh proposed the following Equation (4) to calculate the Modal Accessibility Gap (MAG) between private car and public transit modes [37].

(4) $${\mathrm{MAG}}_{j}{}^{C-P}=\bar{A_{j}^{C}}-\bar{A_{j}^{P}}\forall j\in J$$

In Equation (4), $\bar{A_{j}^{C}}$ represents the private car mode accessibility normalization value, $\bar{A_{j}^{P}}$ represents the public transit mode accessibility normalization value, ${\mathrm{MAG}}_{j}{}^{C-P}$ represents the modal accessibility gap value between the private car mode and the public transit mode, and J represents the unit set. Normalization can be further expressed as Equation (5).

(5) $$\bar{A_{j}^{M}}=\frac{A_{j}^{M}{-\min}_{j\epsilon J}A_{j}^{M}}{\max_{j\epsilon J}A_{j}^{M}{\;-\;\min}_{j\epsilon J}A_{j}^{M}}\forall j\in J$$

In Equation (5), $\bar{A_{j}^{M}}$ represents the normalized value of accessibility in M transportation mode, min and max represent the minimum and maximum values, and J represents the unit set.

## 3. Results

### 3.1. Determination of Time Threshold t_0_

**Figure 5:**
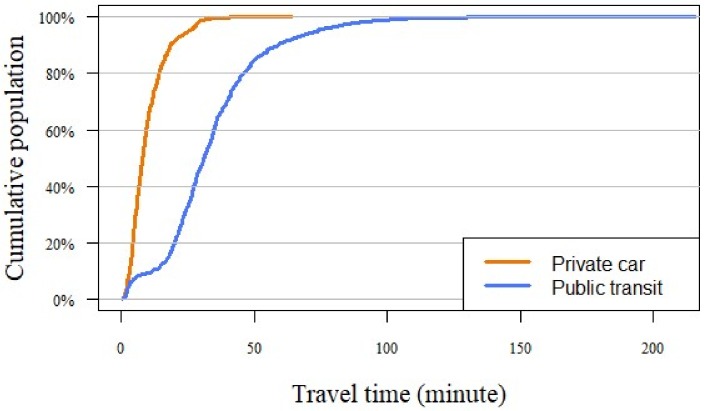
Cumulative proportion of residents accessing the closest general hospital within a certain travel time (Private car: orange line, Public transit: blue line).

According to Section 2.3, the determination of the time threshold *t_0_* is the key to the 2SFCA method. Our model determined the parameter *t_0_* through the following two steps. First, we plotted the relationship between the increase of travel time and the cumulative number of residents using the two modes of transportation to reach the closest hospital, as shown in Figure 5. Next, to ensure that most residents can access at least one hospital by any mode of transportation, we took the travel time of 70%, 80%, and 90% of the residents to their closest hospital as a collection of threshold *t_0_*, the specific values of which are shown in Table 1.

As seen in Table 1, 70% of residents access the closest hospital by car within 12 min. By contrast, 70% of residents access the closest hospital by public transit within 40 min.

### 3.2. Distribution of and Difference in Accessibility to General Hospitals by Private Car and Public Transit Modes

**Figure 6:**
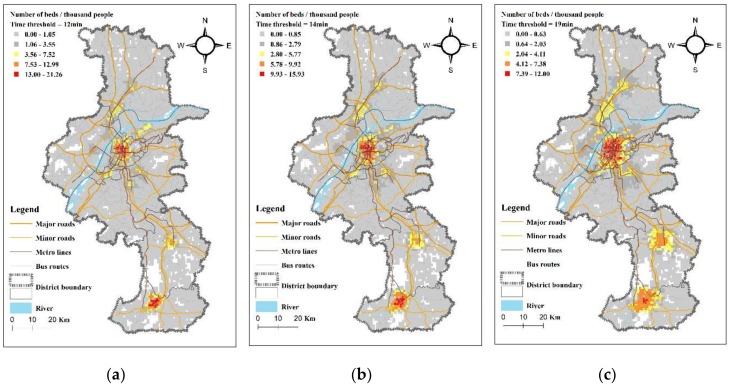
Accessibility of general hospitals in Nanjing by private car mode. The time thresholds were determined to be (**a**) 12 min, (**b**) 14 min, and (**c**) 19 min, according to the travel time of 70%, 80%, and 90% of residents accessing the closest hospital.

**Figure 7:**
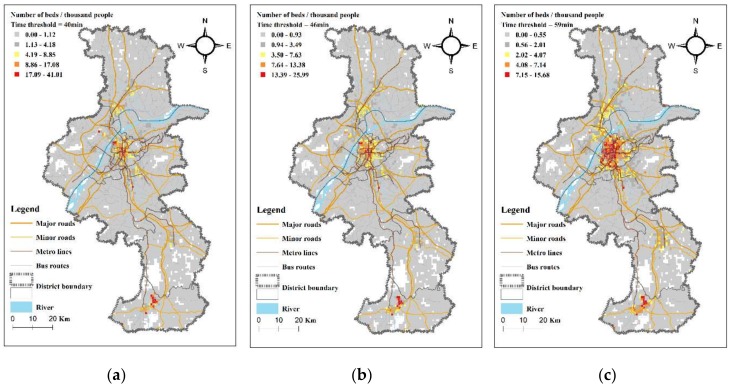
Accessibility of general hospitals in Nanjing by public transit mode. The time thresholds were determined to be (**a**) 40 min, (**b**) 46 min, and (**c**) 59 min, according to the travel time of 70%, 80%, and 90% of residents accessing the closest hospital.

Using the Ga2SFCA method to calculate the accessibility of each cell in the study area, the scores (supply and demand ratio) and the spatial distribution of accessibility to the general hospitals in Nanjing by private car and public transit modes were obtained. As shown in Figure 6 and Figure 7, the “Jenks natural breaks classification” method is used to classify the accessibility score into five levels in ArcGIS [38], and each cell is shaded with a different color according to the level. The accessibility score represents the number of beds in general hospitals per thousand residents (the white area in Figure 6 and Figure 7 reflects a unit with no population, and thus the accessibility is not calculated).

From Figure 6 and Figure 7, we see that the Ga2SFCA accessibility scores for the private car mode and the public transit mode have similarities and differences. Because the population and general hospitals in Nanjing are mostly concentrated in the main urban areas (Gulou, Xuanwu, Qinhuai, and Jianye Districts), the location distribution of general hospitals and accessibility distribution are very similar. Specifically, the main urban area is a concentration area of high accessibility, and the areas with poor accessibility are distributed along the edge of the city and the areas away from the hospital. Interestingly, the accessibility of residents in some areas of Jiangning District is high for both modes of transportation because the hospitals in the area are concentrated and the population density is small, resulting in more hospital beds per capita. The accessibility distribution of the two transportation modes also shows a similar situation that changes with the increase of the time threshold. As the time threshold increases, the accessibility distribution shape changes from a multi-peak distribution to a circular diffusion centered on the main urban area: the longer the time threshold, the more uniform the overall accessibility distribution. On the other hand, the different transportation modes have different characteristics of diffusing accessibility. For public transit, high-accessibility grids distribute along the bus routes and metro lines, especially distributing along the axial direction of the metro lines. For private cars, high-accessibility grids distribute along the road network, with especially high accessibility values concentrated in the urban expressway coverage areas.

**Figure 8:**
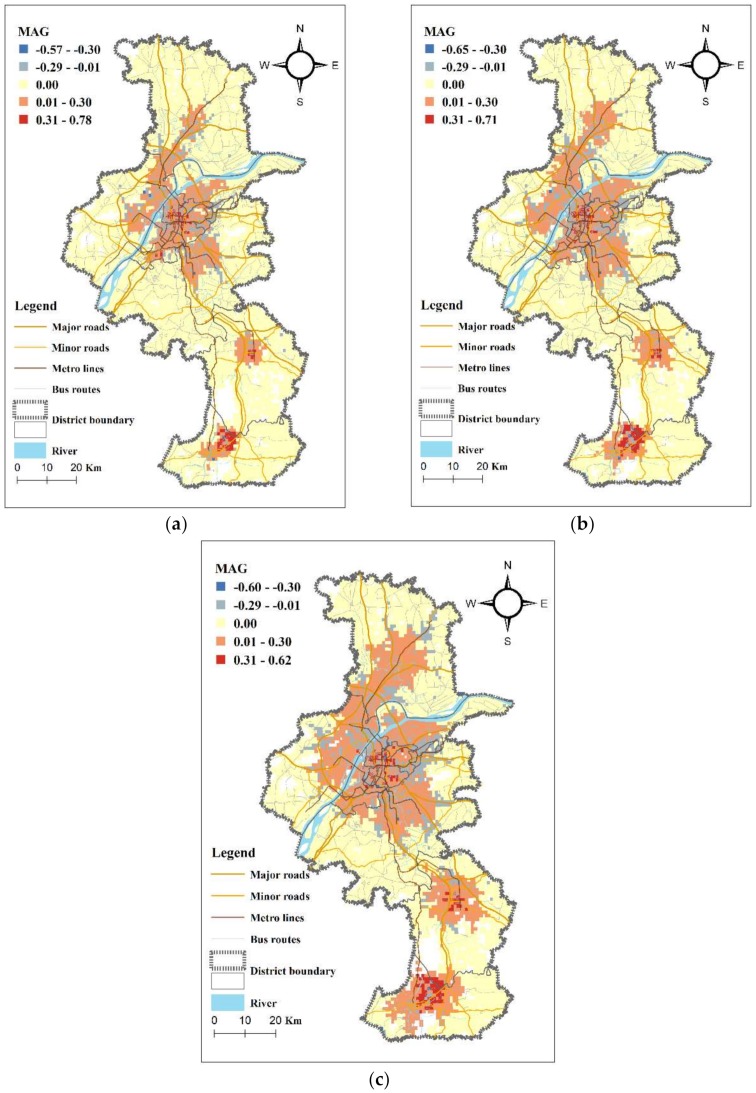
The Modal Accessibility Gap (MAG) to general hospitals between private car and public transit modes under different time thresholds: (**a**) 70%, (**b**) 80% and (**c**) 90%.

To further demonstrate the differences in distribution of spatial accessibility to general hospitals between the two modes of transportation, we normalized the Ga2SFCA accessibility scores of the private car and public transit modes to obtain the Modal Accessibility Gap (MAG). The MAG value is calculated using Equation (4), and the normalization value is calculated using Equation (5). The MAG distribution is shown in Figure 8, in which red cells indicate where the accessibility of the private car mode to general hospitals is greater than the accessibility of the public transit mode at a certain time threshold, and vice versa in blue cells. Additionally, the darker the color, the greater the accessibility gap. There is no accessibility gap in Yellow cells. Visually, most of the area has greater or equal accessibility of the private car mode to general hospitals than that of the public transit mode, under all the three thresholds. The higher the threshold, the larger the red area. Blue cells, where the accessibility of the public transit mode is higher than that of the private car mode, are mostly distributed in peripheral regions outside the red centered areas. Only a few blue cells are scattered right along metro or bus routes in city center. Thus it can be seen that, in the central area where general hospitals are densely distributed, public traffic may not contribute to the access to medical services as much as we thought. For a short journey to a nearby hospital, people would prefer private car than public transit because transferring time cost, e.g., the walk to bus stations and the wait for bus, is considerable compared with the entire travel time. On the contrary, public transit plays a role in areas far from hospitals along metro lines and bus routes, especially along express bus routes (those have longer routes and less stops).

### 3.3. Sensitivity Analysis of Spatial Accessibility to General Hospitals with Different Time Thresholds

To accurately describe the changes in the accessibility to general hospitals with the increase of the time threshold, we calculated the accessibility values of the two modes of transportation for the time thresholds from Table 1. The results of this analysis are shown in Table 2. First, the data show that for the private car mode, the average accessibility values of hospitals within the 12 min, 14 min, and 19 min time thresholds are 0.65, 0.54, and 0.49, respectively, which means that 0.65, 0.54, and 0.49 beds, respectively, can be provided per thousand people. Similarly, for the public transit mode, the average accessibility values of hospitals within the 40 min, 46 min, and 59 min time thresholds are 0.46, 0.43, and 0.42, respectively, which means that 0.46, 0.43, and 0.42 beds, respectively, can be provided per thousand people. The average accessibility scores of the two modes of transportation within the limited time are much lower than 3.05 per thousand people (existing total number of hospital bed/total number of people; see Section 2.2.2 for details). Second, within the limits of all thresholds, the average accessibility value of the private car mode is generally higher than that of the public transit mode, and the coefficient of variation is reversed. However, the coefficient of variation is still at a strong variability level (value greater than 2.8), which indicates that the distribution of general hospital accessibility is significantly regionally unbalanced. Third, as the time threshold increases, the average of the accessibility scores of the two modes of transportation decreases, the standard deviation decreases, which indicates that accessibility displays an increasingly balanced distribution over time.

### 3.4. Accessibility Classification by Private Car and Public Transit Modes

**Figure 9:**
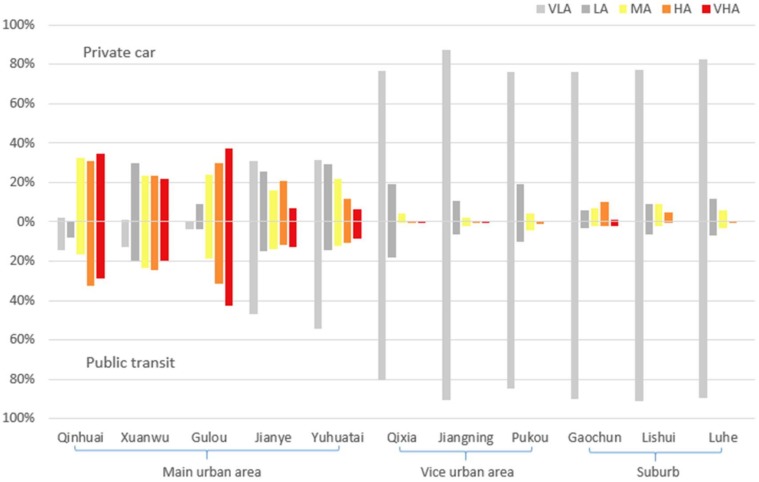
Area proportion in different levels of private car and public transit accessibility to general hospitals in 11 districts. Accessibility levels are classified according to the Jenks natural breaks method.

On the basis of our thematic maps of the accessibility distribution and the modal accessibility gap distribution using multi-time thresholds with the two modes of transportation, it is evident that the current state of distribution of accessibility to general hospitals in Nanjing is uneven. To ensure our research results are relevant to scholars and policymakers, we used the Jenks natural breaks classification method to classify general hospital accessibility into five levels in ArcGIS [38] (Very High Accessibility: VHA; High Accessibility: HA; Medium Accessibility: MA; Low Accessibility: LA; Very Low Accessibility: VLA). Then, we employed spatial overlay analysis to calculate the unit accessibility classification level and the corresponding population of the administrative districts of Nanjing. Using the time threshold corresponding to 90% of residents accessing the closest hospital, the results of the accessibility classification by private car and public transit modes are shown in Figure 9 (Table 1).

The results show that the area with VHA, HA, and MA levels of general hospital accessibility for both modes of transportation in the main urban area (including Gulou, Xuanwu, and Qinhuai Districts) is quite large. Most area in the three districts is in accessibility equal to or higher than medium level. For the other two districts in main urban area, Jianye and Yuhuatai districts, area with VLA and LA accessibility is larger than that with VHA and HA accessibility, especially in public transit mode. For urban fringe areas such as Qixia, Luhe, Pukou, Jiangning, Gaochun, and Lishui Districts, most of the population is within the VLA accessibility level. The primary reasons for these results are that the main urban area has a large distribution density of general hospitals and that the transportation environment is very convenient.

## 4. Discussion

The proximity of residence to the public service facility is usually represented by travel time or travel distance [17]. For medical service accessibility, travel time is more suitable than travel distance in measuring the spatial impedance between residents and medical institutions, especially for sudden illness, and travel distance is not a critical feature for public transport. Therefore, our model used travel time as an important parameter for measuring accessibility. Based on the results of this study, we can see that although we set the time threshold *t_0_* corresponding to 90% of residents accessing the closest hospital, a significant percentage of residents in the non-main urban areas are at the VLA accessibility level by both private car and public transit modes. The accessibility to general hospitals is indeed inadequate and regionally unbalanced in Nanjing, and the main reason for this result is the unbalanced allocation of medical service facilities. We think that the allocation of medical resources should take into account the convenience of public transit, such as the proximity to stations (e.g., metro or bus station), which is beneficial to the segments of the population with disadvantaged mobility, rather than only considering the car mode.

The critical implications for urban planning to improve the accessibility of the city’s overall hospitals are summarized as follows. First, improving accessibility to general hospitals for residents by improving the convenience of public transit in cities is a major concern for urban planners. Accessibility to general hospitals in the non-main urban area of Nanjing is generally poor. Therefore, improving public transit in these urban fringe areas is the key to improving accessibility to general hospitals. In addition, improving the urban bicycle riding environment could help reduce the gap between public transit accessibility and private car accessibility. Previous studies have shown that the effective access of shared or public bicycles near public transit can improve efficiency to a certain extent [36,39]. Second, based on the results from Jiangning District, accessibility for residents using private cars is very sensitive to the grade and layout density of the road. Therefore, improving the construction of urban expressways (highway, ring road, etc.) is an important way to improve accessibility to general hospitals for those traveling by private cars. In addition, in road-dense areas such as Jiangning District, construction of additional medical service institutions such as medical emergency stations may be warranted. Finally, the contradiction in the accessibility to medical and health services in Nanjing is mainly due to the imbalance of medical and health resources allocation. Therefore, another key way of improving accessibility to general hospitals is a spatial distribution of medical service facilities that could better cut down the inequity between areas and transportation modes. More importantly, we found that the accessibility gap between transportation modes is significant, and thus the allocation of medical resources should consider the accessibility differences of various transportation modes.

This study has some limitations that we hope to improve in future work. First, we determined the time threshold based on travel time spent by residents accessing the closest hospital, but residents do not always choose the closest hospital as their medical institution. Therefore, making *t_0_* more reasonable requires relevant survey data support. Second, although the Baidu Map navigation service provides real-time dynamic navigation information, we have not collected the impact of peak traffic on residents’ access to medical services. We also did not compare the navigation accuracy and efficiency of Baidu Maps with other internet maps. Finally, due to reasons such as privacy, data accuracy, and data sufficiency, we did not collect data on the age distribution of residents, the disease distribution of residents, and transportation mode preferences. Therefore, we could not evaluate specific residents’ accessibility to general hospitals and establish the regression relationship between these factors and accessibility. In future studies, we will integrate multiple transportation patterns into the 2SFCA model and collect travel time data during peak traffic to establish a multi-period, multi-mode, high-resolution space–time accessibility model.

## 5. Conclusions

This study was an exploration of urban general hospital accessibility at a higher resolution with the support of internet maps and social media data. In this study, Ga2SFCA was used to calculate the accessibility (supply and demand ratio) distribution and variation of general hospitals in different time thresholds using private car and public transit modes in Nanjing City. The supply was presented by number of beds, and the demand was presented by gridded population derived with social media data, which is updated and precisely delineates the distribution of urban residents compared with community-based study units. With the “door-to-door” approach from Baidu Maps’ navigation service, the obtained travel time is more reliable and updated compared with simulating car travels on road network. Travel time for different transportation modes also better fits the choices of urban residents, and of different sections of population for medical services.

Our research shows that, regardless of transportation mode, general hospital accessibility has a high value distribution in the central urban area and a low value distribution in the non-central urban areas. When the time threshold is lower, the accessibility distribution presents a multi-center annular structure centered on the general hospitals and decreasing toward their periphery; as the time threshold increases, the accessibility distribution pattern changes from a multi-peak distribution to a circular diffusion centered on the main urban area and overall accessibility tends to be better. Seeing from the statistics of accessibility and the comparison among 11 districts varying from main urban area to suburbs, the accessibility to general hospitals in Nanjing is significantly regionally unbalanced in both travel modes.

By mapping the MAG between private car mode and public transit mode, it is found that private car is superior to public transit in most area in access to general hospitals, especially in central city. In central area, public traffic may not contribute to the access to medical services as much as we thought. On the contrary, public transit plays a role in areas far from hospitals along metro lines and bus routes.

Since the travel time of the door-to-door approach includes all the stages of travel, our model could further improve the grid population as residential locations to measure the accessibility by the door-to-door approach. Therefore, the model could be used not only as a guide for accessing general hospitals by different sections of the population, but also to analyze other special situations of medical demands for different segments of the population (e.g., population groups with mammogram screening demand, people with mental or cancer health conditions, or the elderly and minors). Our findings are useful to healthcare administrators and relevant decision makers to assist with the allocation of healthcare resources and the development of health-related policies. In addition, our model could be used as a reference for other cities like Nanjing in developing countries.

## Figures and Tables

**Table 1:** Travel time of a cumulative percentage of residents reaching the closest hospital by different mode of transportation.

Mode of Transportation	Cumulative Resident Percentage	Closest Time
Private car	70%	12 min
	80%	14 min
	90%	19 min
Public transit	70%	40 min
	80%	46 min
	90%	59 min

**Table 2:** Hospital accessibility statistics of private car and public transit.

Transportation Mode	Time Threshold	Minimum Accessibility	Maximum Accessibility	Accessibility Average	Standard Deviation	Coefficient of Variation
Private car	12 min	0	21.26	0.65	1.86	2.86
	14 min	0	15.93	0.54	1.68	3.11
	19 min	0	12.00	0.49	1.54	3.14
Public transit	40 min	0	41.01	0.46	2.03	4.41
	46 min	0	25.99	0.43	1.74	4.05
	59 min	0	15.68	0.42	1.44	3.43
